# The vascular–immune interface in breast cancer: normalization strategies, combination therapy, and biomarkers

**DOI:** 10.3389/fonc.2026.1962446

**Published:** 2026-10-22

**Authors:** Yujing Zhang, Cai Yulan, Kaiguo Long, Qi Wang, Chuyu Liu, Feng Zeng

**Affiliations:** 1 Thyroid and Breast Surgery, The Second Affiliated Hospital of Zunyi Medical University, Zunyi, Guizhou, China; 2 Department of Endocrinology and Metabolism, The Second Affiliated Hospital of Zunyi Medical University, Zunyi, Guizhou, China

**Keywords:** anti-angiogenic therapy, biomarkers, breast cancer, immunotherapy, tumor vasculature, vascular normalization

## Abstract

Immune checkpoint inhibitors have improved outcomes in selected patients with breast cancer, particularly those with triple-negative disease, but most tumors remain refractory. Abnormal tumor vasculature is one contributor to this resistance. Disorganized, hyperpermeable vessels produce heterogeneous perfusion, hypoxia, acidosis, and elevated interstitial pressure; these changes impede drug delivery and lymphocyte trafficking while favoring regulatory T cells, myeloid-derived suppressor cells, and dysfunctional antigen presentation. Anti-angiogenic therapy can transiently reverse part of this phenotype. When dose and timing are appropriate, a vascular-normalization window improves perfusion and oxygenation, restores endothelial adhesiveness, and creates conditions in which chemotherapy and immune checkpoint blockade may work more effectively. This framework is biologically compelling, but clinical evidence in breast cancer remains uneven: activity has been reported in several early-phase combination studies, whereas large trials of bevacizumab-containing regimens have not consistently improved overall survival. Candidate biomarkers—including VEGF/VEGFR signaling, angiopoietin–Tie2 activity, PD-L1, high endothelial venules, tertiary lymphoid structures, circulating cytokines, and spatial patterns of immune exclusion—remain exploratory and are limited by assay variability and temporal heterogeneity. This narrative review examines the vascular – immune interface across breast cancer subtypes, distinguishes mechanistic rationale from clinical proof, and identifies priorities for biomarker-guided trials designed to define dose, sequence, and the normalization window.

## 1 Introduction

Breast cancer comprises biologically distinct diseases, including hormone receptor-positive/human epidermal growth factor receptor 2 (HER2)-negative, HER2-positive, and triple-negative breast cancer (TNBC). These subtypes differ in immune contexture, angiogenic dependence, and treatment sensitivity. Immune checkpoint inhibitors (ICIs) have established a role in selected patients with TNBC, but responses are not uniform and activity in other subtypes remains limited. Anti-angiogenic therapy (AAT) has also produced strong preclinical effects yet inconsistent clinical benefit in breast cancer. These observations raise a central question: can tumor vessels be treated not only as nutrient conduits but also as regulators of antitumor immunity (1–4)?

Most AATs inhibit vascular endothelial growth factor (VEGF) signaling. Excessive or prolonged vascular pruning can worsen hypoxia, whereas appropriately dosed treatment may transiently normalize vessel structure and function, improve perfusion, and facilitate immune-cell trafficking (1, 4, 5). The therapeutic objective is therefore not maximal vessel ablation, but controlled remodeling of the vascular–immune interface.

Preclinical studies support synergy between vascular normalization and programed cell death protein 1 (PD-1)/programed death-ligand 1 (PD-L1) blockade, and early-phase trials have tested AAT with ICIs, chemotherapy, or poly(ADP-ribose) polymerase (PARP) inhibition (1, 3, 6). However, these regimens differ substantially in tumor subtype, line of therapy, anti-angiogenic mechanism, and dosing. Their results should therefore be interpreted as regimen-specific evidence rather than proof of a uniform class effect.

Patient selection is an unresolved problem. CD8-positive T-cell infiltration, PD-L1 expression, VEGFR2-related measures, circulating angiogenic factors, and spatial immune features have all been proposed as biomarkers, but few have undergone prospective validation for an AAT-containing regimen (1, 2, 6).

This narrative review evaluates how abnormal vessels create physical and molecular barriers to immunity, how immune cells reciprocally remodel the vasculature, and how these mechanisms inform combination therapy. We distinguish biological plausibility, exploratory clinical signals, and validated evidence, and propose priorities for biomarker-guided trials in breast cancer. The article provides a critical narrative synthesis rather than a narrative review or meta-analysis.

## 2 Molecular drivers of abnormal tumor angiogenesis in breast cancer

Physiological angiogenesis is a tightly regulated, multi-step process essential for tissue development, homeostasis, and repair—characterized by precisely coordinated endothelial cell activation, proliferation, migration, tube formation, and vascular maturation, culminating in the establishment of stable, hierarchically organized, and functionally competent blood vessels (7). In contrast, tumor angiogenesis is a pathological, dysregulated adaptation in which malignant cells hijack angiogenic signaling to drive persistent, aberrant neovascularization. This process not only fuels tumor growth and metabolic expansion but also actively promotes local invasion and distant metastasis by compromising vascular integrity, enhancing permeability, and fostering an immunosuppressive microenvironment (**Figure 1**) (8).

**Figure 1:**
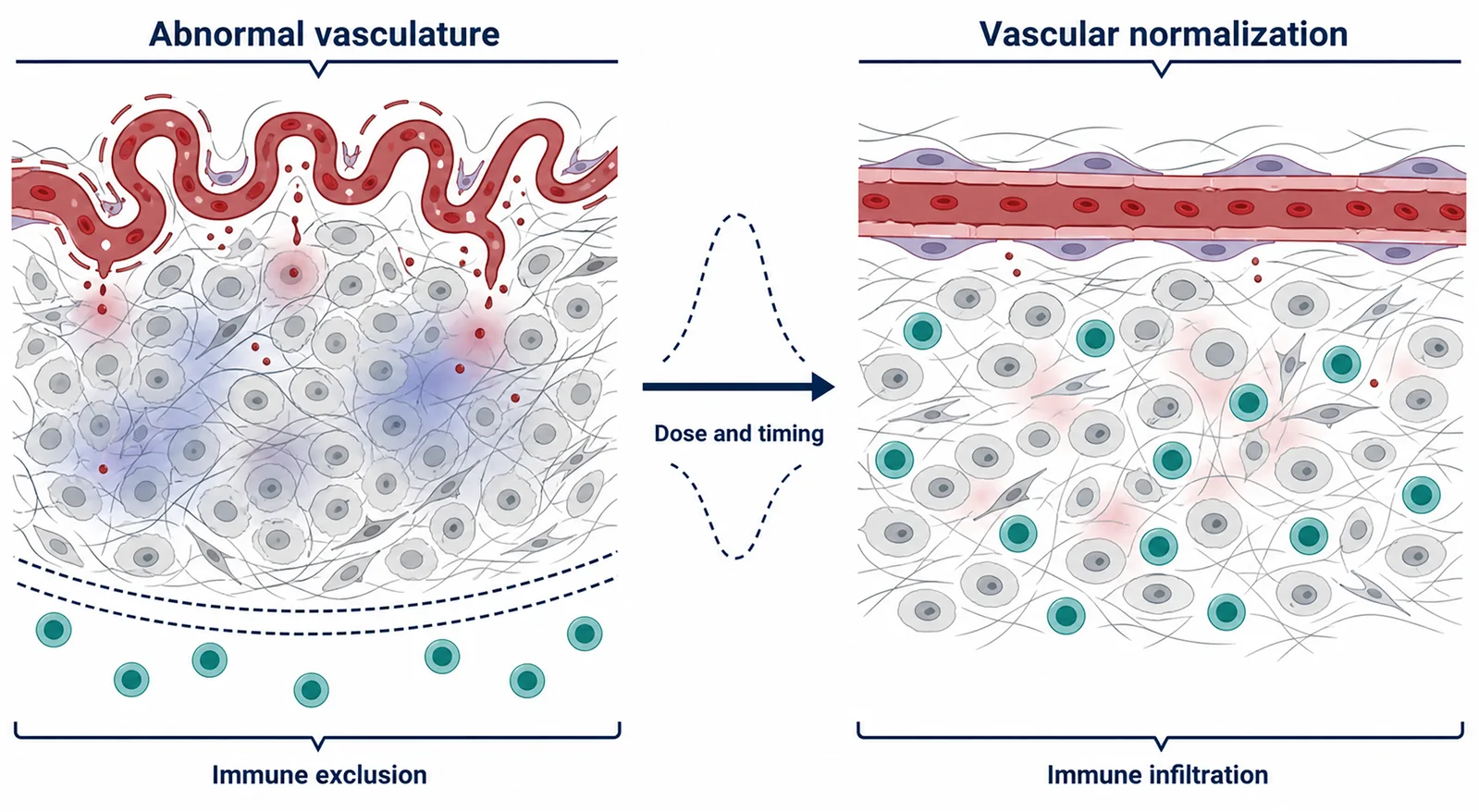
Vascular dysfunction and the immune-excluded tumor microenvironment. Disorganized, hyperpermeable vessels produce heterogeneous perfusion, high interstitial pressure, and hypoxia, limiting drug and lymphocyte delivery and favoring suppressive myeloid cells and regulatory T cells. During a dose- and time-dependent normalization window, improved perfusion and endothelial function may facilitate immune-cell trafficking and increase the activity of anti-angiogenic therapy combined with immune checkpoint inhibitors.

### 2.1 Core signaling pathways driving pathological angiogenesis

During early tumor progression, rapid malignant cell proliferation induces localized hypoxia and nutrient deprivation, triggering extensive transcriptional reprogramming—including upregulation of hypoxia-inducible factors (HIFs) and pro-angiogenic genes—within tumor cells. In response, tumors secrete a coordinated cocktail of pro-angiogenic factors (e.g., VEGF-A, FGF2, PDGF-BB), initiating the “angiogenic switch”: a critical transition in which dormant vasculature is co-opted and activated, leading to endothelial tip-cell selection, sprouting angiogenesis, and directional vessel growth toward the tumor mass (9, 10). Although these newly formed vessels partially alleviate hypoxia and deliver nutrients, they are structurally and functionally abnormal—exhibiting leakiness, poor perfusion, and immature basement membranes—and thereby foster a permissive microenvironment that supports breast cancer cell survival, epithelial–mesenchymal transition (EMT), local invasion, and intravasation into the systemic circulation (7, 11, 12). Consequently, pathological neovascularization not only accelerates primary tumor expansion but also provides both physical conduits and molecular niches essential for hematogenous dissemination and metastatic colonization (13).

Importantly, tumor angiogenesis in breast cancer is not a transient compensatory response but a sustained, dysregulated process driven by a multilayered network of molecular signals, heterogeneous cellular interactions, and dynamic microenvironmental cues. Within this regulatory landscape, vascular endothelial growth factor (VEGF) occupies a central position: its overexpression and sustained signaling activation are consistently associated with aggressive clinicopathological features—including higher tumor grade, lymph node metastasis, and reduced recurrence-free survival—establishing VEGF as a validated prognostic biomarker and a principal therapeutic target (14–16). Nevertheless, accumulating preclinical and clinical evidence demonstrates that tumor angiogenesis in breast cancer is orchestrated through both VEGF-dependent and VEGF-independent pathways—including FGF, Angiopoietin–Tie2, Notch, and hypoxia-driven alternative mechanisms—highlighting the intrinsic redundancy, plasticity, and adaptive resilience of the tumor vasculature (11, 17, 18).

Breast tumors can obtain a blood supply through several routes, and this distinction matters therapeutically. Sprouting angiogenesis is VEGF dependent and remains the canonical target of bevacizumab and VEGFR tyrosine kinase inhibitors. Vessel co-option, by contrast, utilizes pre-existing host vessels and may contribute to resistance to anti-angiogenic therapy, particularly in metastatic sites such as the liver (19) program. Intussusceptive growth, vasculogenic mimicry, and recruitment of endothelial progenitor-like populations provide additional routes. These processes can coexist within one lesion, so a reduction in microvessel density does not necessarily mean that perfusion has been eliminated. It may instead indicate selection for a less VEGF-dependent vascular phenotype (7–9, 20). This heterogeneity offers one explanation for the frequent divergence between impressive xenograft responses and modest clinical benefit. Subcutaneous models are often dominated by rapidly sprouting vessels, whereas human breast cancers evolve over years, contain extensive stromal remodeling, and may occupy organs in which co-option is readily available. Future studies should therefore define the mode of vascularization rather than treating all CD31-positive structures as equivalent.

Angiogenic dependence also varies among breast cancer subtypes and disease sites. TNBC commonly exhibits hypoxia, necrosis, inflammatory signaling, and high immune-cell density, creating a plausible setting for vascular–immune intervention. HER2-positive tumors can show intense growth-factor signaling but are also shaped by HER2-directed treatment, which changes both tumor-cell survival and immune recruitment. Hormone receptor-positive tumors are often less inflamed and may rely more heavily on endocrine and stromal programs. Metastases add another layer: lung and liver lesions may co-opt native sinusoids or capillaries, whereas brain metastases confront a specialized neurovascular unit. Consequently, the same anti-angiogenic dose may normalize one lesion, over-prune another, and leave a co-opted metastasis largely unaffected. Clinical trials that pool subtypes and anatomical sites without vascular stratification risk averaging biologically distinct responses (19, 21–23).

In the subsequent sections, we summarize the key VEGF-dependent and VEGF-independent signaling pathways that drive pathological angiogenesis in breast cancer, establishing a mechanistic framework for understanding vascular dysregulation and its implications for disease progression and therapeutic resistance.

#### 2.1.1 Hypoxia–HIF–VEGF signaling

Hypoxia is a major upstream driver of angiogenesis. Stabilized HIF-1α and HIF-2α increase VEGFA and other pro-angiogenic programs, while additional transcriptional and post-transcriptional regulators can sustain this response as oxygen availability fluctuates (24). The resulting vessels remain poorly organized and inefficiently perfused. Hypoxia also favors TGF-β and IL-10 signaling, recruitment of regulatory T cells and myeloid-derived suppressor cells (MDSCs), and expression of inhibitory ligands, thereby linking angiogenic stress to immune dysfunction. These mechanisms are context dependent, and evidence for individual regulatory nodes should not be generalized across all breast cancer subtypes.

The HIF response is spatially and temporally heterogeneous. Chronic hypoxia develops in regions remote from functional vessels, whereas cycling hypoxia follows intermittent vessel collapse and reperfusion. These states do not produce identical transcriptional outputs. HIF-1α often dominates acute glycolytic adaptation, while HIF-2α can support longer-term endothelial and tumor-cell programs; both interact with oncogenic and metabolic pathways (25, 26). Reoxygenation can further increase reactive oxygen species and inflammatory signaling. A single tissue section therefore provides only a snapshot of a moving oxygen landscape. This limitation is important when hypoxia markers are proposed as predictors of a normalization window: marker expression may reflect prior oxygen exposure rather than current perfusion. Longitudinal imaging and repeated blood or tissue sampling are more informative than baseline staining alone (24, 27, 28).

#### 2.1.2 Stromal–endothelial crosstalk

Stromal cells provide VEGF-independent routes to endothelial activation. Cancer-associated fibroblasts can engage YAP1-, STAT3-, Wnt-, NF-κB-, and ERK-related programs, whereas platelets supply integrin- and TGF-β-dependent signals that support vascular remodeling (18). Matrix deposition and perivascular inflammation further impede lymphocyte access. Because several of these pathways also regulate antigen presentation and myeloid polarization, stromal signaling can maintain both angiogenesis and immune suppression after VEGF blockade.

Cancer-associated fibroblasts are themselves heterogeneous. Myofibroblastic, inflammatory, and antigen-presenting-like states differ in their spatial distribution and secretory programs, and their contribution to angiogenesis cannot be inferred from a single marker such as α-smooth muscle actin (29). Perivascular fibroblasts can alter basement-membrane composition and vessel stiffness, while inflammatory fibroblasts supply IL-6-family cytokines, CXCL12, and growth factors that shape myeloid recruitment (30). Some stromal programs may restrict tumor growth or preserve tissue architecture, so indiscriminate fibroblast depletion can be counterproductive. A more useful strategy is to identify the particular stromal circuit that sustains endothelial dysfunction or immune exclusion in a given tumor. This requires spatially resolved measurements of fibroblast state, vessel phenotype, and immune-cell location rather than bulk expression averages (18, 27, 31).

#### 2.1.3 Metabolic regulation of VEGF signaling

Endothelial metabolism is not merely a consequence of angiogenesis. PFKFB3-dependent glycolysis promotes endothelial proliferation and may reinforce VEGF signaling, while von Willebrand factor (vWF) – PI3K – AKT and FGFR1-related programs link receptor signaling to mitochondrial and glycolytic adaptation (32–35). Lactate accumulation, extracellular acidification, and persistent oxygen gradients impair lymphocyte proliferation and effector function (36). Metabolic interventions may therefore complement vascular targeting, but their therapeutic window in breast cancer remains undefined.

Endothelial metabolic adaptation also helps explain why angiogenic vessels can proliferate in an oxygen-poor environment. Endothelial cells derive much of their ATP from glycolysis, limiting their own oxygen consumption while permitting growth along oxygen gradients. Excess PFKFB3 activity favors sprouting, whereas partial glycolytic inhibition can reduce pathological branching without necessarily destroying established vessels (33, 35). Lactate produced by tumor and stromal cells serves both as a fuel and as a signaling metabolite; it can stabilize pro-angiogenic programs, polarize macrophages, and impair cytotoxic lymphocytes (11). These observations suggest that metabolic normalization could complement VEGF blockade. However, systemic inhibition of glycolysis or mitochondrial function may also compromise activated T cells, which undergo their own metabolic reprogramming. The relevant therapeutic question is therefore not whether metabolism matters, but whether endothelial metabolism can be modulated selectively enough to improve perfusion without suppressing immune effectors (11, 31, 37, 38).

#### 2.1.4 Cancer stemness and vascular resistance

Stemness-associated programs can sustain angiogenic signaling and treatment resistance. ALDH1A1-positive breast cancer cells and IL-6/STAT3-related circuits have been linked to VEGF production, vascular permeability, immune exclusion, and resistance (27). Low-dose AAT can partially reverse these features in experimental models, but the magnitude and duration of normalization depend on tumor context and dose (28).

### 2.2 Heterogeneity and functional specialization of tumor endothelial cells

Tumor endothelial cells exhibit significant heterogeneity, with their molecular and functional characteristics varying according to tissue microenvironment and disease state. Single-cell studies have revealed that tumor endothelial cells can repeatedly adopt several functional states, including apical-like, stem-like, and a highly permeable phenotype characterized by elevated expression of plasma membrane vesicle-associated protein (PLVAP). However, these states do not represent fixed cell types but rather form a continuous spectrum. Geldhof et al. analyzed 26,515 single cells from nine breast cancer patients, including 8,433 endothelial cells, and identified distinct endothelial populations with unique transcriptional programs in malignant versus peritumoral tissues. Notably, a lipid-handling endothelial cell type (LIPEC), characterized by genes related to lipid metabolism and PPAR-γ signaling pathways, was enriched in the peritumoral breast tissue. Predicted receptor-ligand interactions suggest potential communication between endothelial cells and immune cells (39). These findings support the view that endothelial cells in breast tumors are not a uniform population of aberrant vessels, but rather comprise multiple specialized states with distinct metabolic and immune functions, consistent with the broader understanding that tumor endothelial functional states exist as a continuous lineage regulated by tissue context and disease status program.

#### 2.2.1 Tip-like endothelial cells

Tip-like endothelial cells occupy the leading edge of angiogenic sprouts and express markers such as DLL4, CXCR4, KIT, VEGFR2, and NRP1. They sense VEGF gradients, extend filopodia, and direct sprout migration, whereas lumen formation is primarily coordinated by trailing endothelial cells (37). Enrichment of tip-like programs at invasive fronts has been associated with aggressive angiogenesis, although their prognostic value in breast cancer requires independent validation (38).

#### 2.2.2 Stalk-like endothelial cells

Stalk-like endothelial cells follow tip cells, proliferate, and form the vascular lumen. Notch activation by tip-cell DLL4 restrains excessive tip specification and coordinates ordered sprouting; Tie2, HEY1, JAG1, and related programs contribute to stabilization (37). Excess VEGF disrupts the tip–stalk balance and promotes tortuous, hyperbranched networks (11).

#### 2.2.3 PLVAP-positive endothelial cells

PLVAP marks fenestrated, highly permeable endothelial states that are enriched in several tumors and can contribute to leakage, high interstitial pressure, and inefficient delivery (40, 41). Reduced endothelial adhesion molecules may accompany this phenotype, but direct evidence that PLVAP itself causes CD8-positive T-cell exclusion or predicts anti-VEGF resistance in breast cancer remains limited (42). PLVAP should therefore be considered a candidate vascular-state marker rather than a validated predictive biomarker.

Single-cell nomenclature requires caution. Labels such as tip, stalk, venous, arterial, capillary, and PLVAP-positive describe transcriptional similarities to physiological endothelial states; they do not establish stable lineages. Dissociation preferentially loses fragile or poorly perfused cells, and endothelial abundance is low relative to epithelial and immune compartments, making sampling depth a major source of bias. Treatment can also shift gene expression without changing lineage. For example, a cell classified as tip-like before therapy may acquire inflammatory or interferon-responsive features after treatment. Cross-study comparison is further complicated by different tissue-processing methods, marker panels, and clustering thresholds. Although single-cell studies have established an initial endothelial atlas in breast cancer, further refinement will require harmonized sampling across molecular subtypes, primary tumors and metastases, and treatment time points, followed by validation using multiplex imaging or spatial transcriptomics (37, 39, 41–43).

In normal tissues, vascular networks are hierarchically organized, characterized by intact endothelial layers, continuous basement membranes, and adequate pericyte coverage, which together ensure stable blood flow and efficient material exchange (18). In stark contrast, tumor vasculature in breast cancer exhibits marked structural abnormalities, including chaotic architecture, irregular luminal diameters, thin vessel walls, excessive tortuosity, and markedly reduced pericyte and smooth muscle cell coverage. These alterations compromise vascular integrity and increase permeability, resulting in widespread leakage and hemodynamic instability (18, 24). Collectively, these features provide the structural basis for impaired perfusion, elevated interstitial fluid pressure, heterogeneous drug delivery, and immune cell exclusion (43).

### 2.3 Aberrant structure and function: from hypoperfusion to immune exclusion

In contrast to the well-organized microcirculation of normal tissues, neovessels in breast tumors exhibit irregular lumens and disorganized branching patterns, forming heterogeneous vascular networks with variable diameters (43). Incomplete basement membrane formation and insufficient pericyte coverage compromise vessel wall integrity, rendering tumor vessels fragile, hyperpermeable, and prone to collapse (44). Consequently, plasma components readily extravasate into the tumor stroma, elevating interstitial fluid pressure and impairing the delivery of oxygen, nutrients, and therapeutic agents, thereby contributing to spatially heterogeneous drug distribution and therapeutic resistance (**Figure 2**) (44). In this context, vasculogenic mimicry deserves special mention: breast cancer cells can form matrix-rich, vessel-like channels without endothelial lining. This phenomenon is associated with invasive disease and may provide alternative perfusion pathways, although its role in immune exclusion and resistance to anti-angiogenic therapy remains incompletely understood (45).

**Figure 2:**
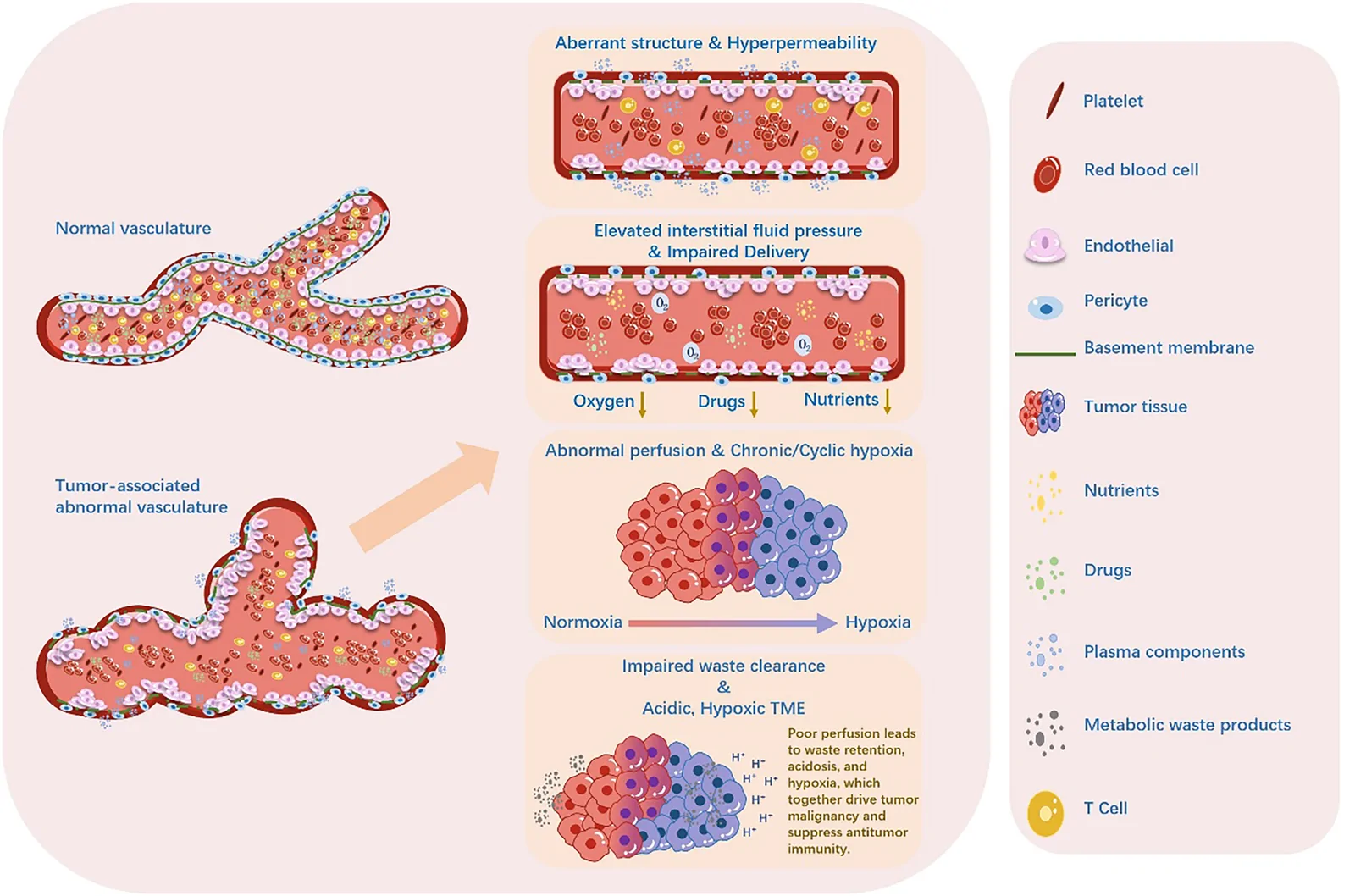
Structural and functional abnormalities of breast tumor vessels. Compared with normal vasculature, tumor vessels show irregular branching, incomplete endothelial and pericyte coverage, hyperpermeability, elevated interstitial pressure, heterogeneous delivery of oxygen and drugs, impaired waste clearance, acidosis, and chronic or cycling hypoxia.

Importantly, abnormal tumor vasculature not only disrupts material exchange but also constitutes a critical physical and functional barrier to antitumor immunity. Impaired perfusion and hypoxia simultaneously restrict immune cell trafficking into the tumor parenchyma and suppress immune effector function, thereby collectively fostering immune escape (43). Consequently, breast tumors are characterized by discontinuous endothelial junctions, fragmented basement membranes, and impaired vascular maturation—features that collectively drive dysfunctional perfusion and sustained hypoxia (9).

Abnormal angiogenesis thus constitutes both a prerequisite for tumor growth and a major obstacle to effective therapy, forming the biological rationale for vascular normalization strategies. Clinically, microvessel density (MVD) serves as a key indicator of the extent of tumor angiogenesis. Elevated MVD not only reflects a greater degree of vascular abnormality but is also closely associated with perfusion defects, exacerbated hypoxia, enhanced immunosuppression, and increased therapeutic resistance. MVD has been associated with metastatic risk and poor prognosis in multiple breast cancer cohorts, although its prognostic value depends on methodology, vascular marker selection, scoring system, tumor region sampled, and clinical context. MVD is therefore best regarded as a context-dependent prognostic indicator rather than a universally validated clinical biomarker (46, 47). Collectively, these structural and functional vascular abnormalities create a hypoxic and immunosuppressive microenvironment, providing a mechanistic link between aberrant angiogenesis and immune evasion—a topic that will be further elaborated in the following section.

The measurement of tumor vessels should move beyond density. Microvessel density captures the number of stained vascular profiles but provides limited information about lumen patency, flow direction, permeability, pericyte investment, or regional oxygen delivery. Two tumors with similar vessel density may have very different functional perfusion. More informative composite assessment could combine vessel morphology, pericyte coverage, basement-membrane continuity, perfused-vessel fraction, hypoxia, and the distance between vessels and immune cells. Dynamic contrast-enhanced magnetic resonance imaging, perfusion computed tomography, contrast-enhanced ultrasound, and positron-emission approaches can provide noninvasive surrogates, although each measures a different aspect of flow or permeability. Histological and imaging endpoints should therefore be selected to match the proposed mechanism rather than grouped under the broad label of vascular normalization (43–46, 48, 49).

## 3 Tumor vasculature as a hub for immune regulation

As discussed above, structurally abnormal, poorly perfused tumor vessels establish a hypoxic and immunosuppressive microenvironment that drives tumor progression and therapeutic resistance. Conversely, immune cells are not passive bystanders but actively shape vascular architecture and function. This reciprocal interplay between immune cells and tumor vasculature constitutes a central regulatory axis in breast cancer progression, determining whether vessels remain dysfunctional or undergo normalization (**Figure 3**) (11).

**Figure 3:**
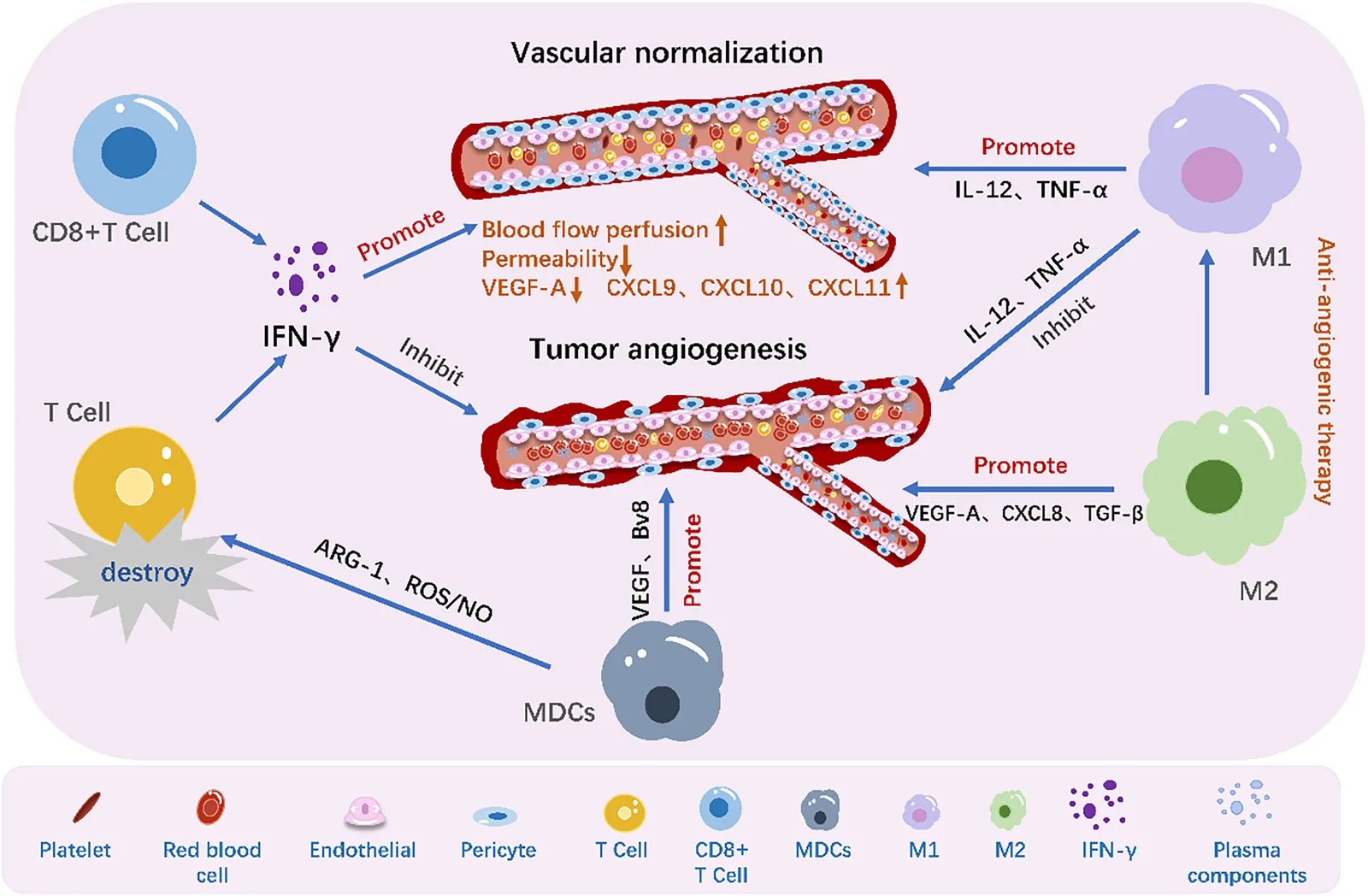
Bidirectional regulation between immune cells and tumor vessels. IFN-γ-producing T cells and pro-inflammatory macrophage states can support vascular maturation, whereas MDSCs and suppressive macrophage states release VEGF, Bv8, CXCL8, TGF-β, and other factors that sustain angiogenesis and immune dysfunction. The balance is context dependent; arrows indicate reported regulatory relationships rather than universal effects. ARG-1, arginase-1; MDSC, myeloid-derived suppressor cell; ROS, reactive oxygen species.

### 3.1 Abnormal vasculature shapes an immunosuppressive microenvironment

Abnormal vessels shape immunity through three connected mechanisms. First, irregular perfusion and high interstitial pressure restrict lymphocyte trafficking. Second, hypoxia and VEGF impair dendritic-cell maturation and endothelial expression of VCAM-1 and ICAM-1 (50, 51). Third, VEGF- and chemokine-rich niches favor regulatory T cells (Tregs), MDSCs, and suppressive macrophages (45, 52, 53). Endothelial PD-L1 and Fas L can add inhibitory or apoptotic signals, while intense VEGFR inhibition may activate compensatory myeloid and neutrophil pathways in some models (54). Hypoxia further amplifies checkpoint expression and suppressive cytokines through HIF-dependent programs (55). Together, these effects create immune exclusion, but the relative contribution of each mechanism differs among tumors.

Endothelial anergy is particularly relevant because lymphocyte trafficking is an active multistep process. Circulating T cells must tether, roll, adhere, crawl, and cross the endothelial barrier before they can reach tumor cells. VEGF, basic fibroblast growth factor, endothelin signaling, and persistent hypoxia can reduce endothelial responsiveness to inflammatory cytokines and suppress E-selectin, ICAM-1, or VCAM-1. At the same time, chemokine presentation at the luminal surface may be inadequate or spatially mismatched. Restoring one adhesion molecule is therefore unlikely to be sufficient if perfusion remains intermittent or the surrounding matrix is impermeable. Conversely, increased intratumoral CD8-positive cells after treatment should not automatically be attributed to vascular normalization; local proliferation, altered retention, and redistribution from adjacent tissue can produce a similar observation. Paired spatial analysis are needed to distinguish entry from expansion (52–55).

### 3.2 Immune cells as regulators of vascular remodeling

#### 3.2.1 T cells: drivers of vascular normalization and immune amplification

Type 1 helper T cells can promote vascular maturation through interferon-γ (IFN-γ), increasing pericyte coverage and improving perfusion in experimental tumors. Better perfusion can, in turn, support further lymphocyte entry, establishing a positive feedback loop between immune activation and vascular normalization (11). Whether this loop is sufficient to predict clinical response remains unknown.

CD8-positive T cells can inhibit angiogenesis through IFN-γ and chemokines such as CXCL9, CXCL10, and CXCL11 (56). Their entry depends on endothelial adhesion pathways, including LFA-1–ICAM-1; VEGF-mediated endothelial anergy can weaken this interaction (57). Tumor endothelium may also express Fas L, which can delete activated Fas-positive lymphocytes at the vascular interface (58). Experimental DLL1 signaling provides another route by which T-cell-derived IFN-γ can support vessel maturation and sensitize resistant tumors to checkpoint blockade (59).

The IFN-γ–vascular relationship is also bidirectional and dose dependent. Moderate, spatially restricted IFN-γ signaling can increase antigen presentation, chemokine expression, and vessel maturation. Sustained high-level signaling, however, may induce counter-regulatory PD-L1, IDO1, and tissue injury. The timing of ICI relative to vascular intervention may therefore determine whether IFN-γ reinforces a productive feedback loop or produces adaptive resistance. Preclinical studies often administer treatment at fixed intervals in synchronized tumors, conditions that are difficult to reproduce clinically. Human trials should incorporate early pharmacodynamic time points rather than waiting until radiographic response assessment. A rise in intratumoral CXCL9/CXCL10, improved perfusion, and movement of CD8-positive cells from perivascular regions into tumor nests would provide stronger evidence for the proposed mechanism than a change in any single marker (47, 56–59).

#### 3.2.2 Tumor-associated macrophages: context-dependent regulators of angiogenesis

Tumor-associated macrophages (TAMs) are functionally diverse and cannot be reduced to a stable M1/M2 dichotomy. Nevertheless, pro-inflammatory macrophage states can release IL-12 and TNF-α and support vascular maturation, whereas suppressive or tissue-remodeling states produce VEGF-A, EGF, FGF, CXCL8, CXCL12, and TGF-β (56, 60). Anti-angiogenic therapy can shift this balance in some models, but macrophage responses vary with dose, tissue context, and treatment duration.

In transgenic breast cancer models, TAMs initially localize within the tumor parenchyma and exhibit low mechanistic target of rapamycin complex 1 (mTORC1) activity. Deletion of the mTORC1 inhibitor TSC1 reprograms TAMs toward a pro-regression phenotype, leading to perivascular redistribution, elimination of PROCR-expressing endothelial progenitors, correction of vascular hyperpermeability, and subsequent induction of tumor hypoxia and cancer cell death (61, 62). Podoplanin-expressing macrophages (PoEMs) promote matrix remodeling, vascular growth, and lymphatic invasion through glycosylation-dependent interactions with lymphatic endothelium-derived galectin-8, correlating with lymph node and distant metastasis in breast cancer patients (63).

Macrophages also regulate vessels through contact-dependent and metabolic mechanisms. DLL4–Notch interactions, VEGF-C/D, IL-1β, and TGF-β-related pathways can alter endothelial sprouting, lymphangiogenesis, and hypoxia signaling (64). These observations identify macrophage–endothelial communication as a therapeutic target, although direct clinical evidence in breast cancer remains preliminary.

Modern macrophage profiling has moved beyond the M1/M2 shorthand. Breast tumors contain interferon-responsive, lipid-associated, hypoxic, proliferating, tissue-resident, and monocyte-derived macrophage states, many of which coexpress conventionally ‘M1’ and ‘M2’ markers. Their function depends strongly on location. Perivascular macrophages can regulate permeability and tumor-cell intravasation, whereas macrophages in necrotic regions encounter different oxygen and metabolic conditions. Tie2-expressing macrophages are of particular interest because they localize near vessels and respond to angiopoietin signals, but their abundance alone does not establish a causal role in resistance. Therapeutic interpretation should therefore combine phenotype, spatial niche, and function. A decrease in total macrophages may be less informative than a shift away from VEGF-, MMP9-, and TGF-β-dominant perivascular states toward antigen-presenting or inflammatory states (60, 61, 64).

#### 3.2.3 Myeloid-derived suppressor cells: a link between angiogenesis and immune escape

MDSCs accumulate in response to tumor- and vessel-derived GM-CSF, G-CSF, CXCL5, and related cues. They release VEGF, Bv8, and MMP9, promoting angiogenesis and matrix remodeling while suppressing T cells through arginase-1, reactive oxygen species, and nitric oxide (65, 66). Their perivascular localization may facilitate tissue entry and local disruption of endothelial barrier function. Thus, MDSCs are both a mediator of resistance and a potential pharmacodynamic readout, but no standardized clinical assay has been established.

Translation of MDSC biology is complicated by inconsistent human definitions. Murine granulocytic and monocytic MDSCs can be characterized functionally, whereas clinical studies often rely on variable combinations of CD11b, CD33, HLA-DR, CD14, CD15, and lineage markers. Cryopreservation and sample processing change the apparent abundance of granulocytic populations. Moreover, neutrophils and immature myeloid cells form a continuum, and suppressive function is not guaranteed by phenotype alone. For biomarker development, standardized whole-blood processing, predefined gating, and a functional or molecular suppression readout are preferable. Serial changes may be more informative than baseline abundance because compensatory myeloid recruitment can emerge after initial VEGF-pathway inhibition. These methodological constraints partly explain why strong preclinical links between MDSCs and AAT resistance have not yet produced a validated patient-selection assay (65).

#### 3.2.4 Dendritic cells at the vascular–immune interface

VEGF suppresses dendritic-cell maturation and antigen presentation, thereby weakening T-cell priming. Some experimental studies have also reported endothelial-like features in immature dendritic or myeloid populations under VEGF-rich conditions, but lineage conversion remains model dependent and should not be regarded as a general mechanism (56). Conversely, mature dendritic cells can release IL-12, IL-18, and type I interferons with anti-angiogenic and immunostimulatory effects. Increased mature dendritic-cell infiltration after bevacizumab has been reported in breast cancer and may contribute to improved immune recruitment (53).

Dendritic-cell function depends on more than cell number. Conventional type 1 dendritic cells are specialized for cross-presentation and are particularly relevant to CD8-positive T-cell priming, whereas other dendritic and monocyte-derived populations have different roles. VEGF-rich and hypoxic environments can impair maturation, migration, and costimulatory activity even when dendritic cells remain detectable by immunohistochemistry. Effective vascular–immune therapy should therefore be expected to improve not only infiltration but also antigen uptake, migration to lymphoid structures, and T-cell priming. Tertiary lymphoid structures (TLSs) may provide a local site for these interactions, but their organization and maturity vary considerably. Studies that report ‘increased dendritic cells’ without defining lineage or activation state should be interpreted cautiously (65, 67).

The vascular–immune interface is therefore reciprocal: vessels regulate immune entry and function, while T cells, macrophages, MDSCs, and dendritic cells influence vessel stability and growth. The balance among IFN-γ, VEGF, MMP9, IL-12, adhesion molecules, checkpoint ligands, and chemokines determines whether the microenvironment favors immune access or exclusion (43, 53, 56).

## 4 Vascular normalization as an immunomodulatory principle of anti-angiogenic therapy

The preceding sections identify a translational question: can partial restoration of vascular structure and function improve drug delivery and antitumor immunity without causing excessive vessel loss?

Jain’s vascular-normalization model provides the relevant framework. Appropriately dosed Anti-angiogenic therapy can temporarily improve endothelial integrity, pericyte coverage, perfusion, and oxygenation, creating a limited period in which partner therapies may gain access to the tumor (59, 68). Because VEGF also alters endothelial cytokines and immune function, normalization is both a hemodynamic and immunological intervention (69). The magnitude and duration of the window vary by agent, dose, and tumor biology.

The dose–response relationship is central to normalization. At low or intermediate exposure, VEGF-pathway inhibition may reduce excessive endothelial signaling, tighten junctions, and improve pericyte association. With stronger or prolonged inhibition, vessel loss can reduce perfusion and increase hypoxia. The optimal range is unlikely to be identical for an antibody, a selective VEGFR2 inhibitor, and a multitarget tyrosine kinase inhibitor, because their pharmacokinetics and off-target effects differ. It may also change over the course of treatment as tumor burden, blood pressure, and compensatory pathways evolve. Fixed maximum-tolerated dosing is therefore not necessarily the most rational approach for an immunomodulatory objective. Biological dose optimization should integrate systemic exposure with a direct or surrogate measure of perfusion and immune trafficking (28, 69, 70).

Normalization may reduce hypoxia-driven immunosuppression and increase the entry and function of T cells, natural killer cells, and dendritic cells (11, 28). In a bevacizumab lead-in study followed by durvalumab, vascular features after bevacizumab differed between non-progressors and progressors, supporting—but not validating—normalization status as a candidate response marker (71). These observations justify serial tissue, imaging, or blood-based measurements in future trials.

No single assay currently defines the start and end of a normalization window. Imaging can be repeated and samples the whole lesion, but changes in contrast transfer may reflect both flow and permeability. Tissue provides cellular resolution but is invasive and vulnerable to spatial sampling error. Blood markers are accessible but may originate from platelets, normal endothelium, immune cells, or multiple disease sites. A practical strategy is triangulation: an early imaging change consistent with improved perfusion, a pharmacodynamic change in a circulating angiogenic marker, and a spatial tissue change showing improved immune entry. Concordance across these domains would be more convincing than any isolated result. Trials should specify the expected direction and timing of each measure before enrollment to avoid *post hoc* selection of a favorable ‘window’ (71, 72).

Anti-angiogenic therapy rarely produces durable control as monotherapy in breast cancer and can worsen hypoxia if dosing exceeds the normalization range (71). Model-specific reports of endothelial PD-L1 induction after AAT also suggest that vascular remodeling may reveal new inhibitory signals (73). The rationale for combination therapy is therefore strongest when dose and sequence are explicitly designed around a measured biological window.

## 5 Rationale and clinical evidence for combination therapy

Anti-angiogenic therapy and immunotherapy address complementary barriers. Vascular remodeling may improve perfusion and lymphocyte entry, whereas ICIs restore function in antigen-experienced T cells. Chemotherapy can add antigen release and immunogenic cell death, provided that vascular injury and marrow suppression do not outweigh these effects (4, 28, 54). This rationale has generated several breast cancer trials, but most clinical studies have evaluated treatment efficacy rather than directly demonstrating vascular normalization. Therefore, clinical activity observed with AAT-containing regimens should not be interpreted as evidence of normalization unless accompanied by pharmacodynamic measurements of vascular structure or function, such as improved perfusion, reduced permeability, increased pericyte coverage, or restoration of endothelial integrity (**Figure 4**; **Table 1**).

**Figure 4:**
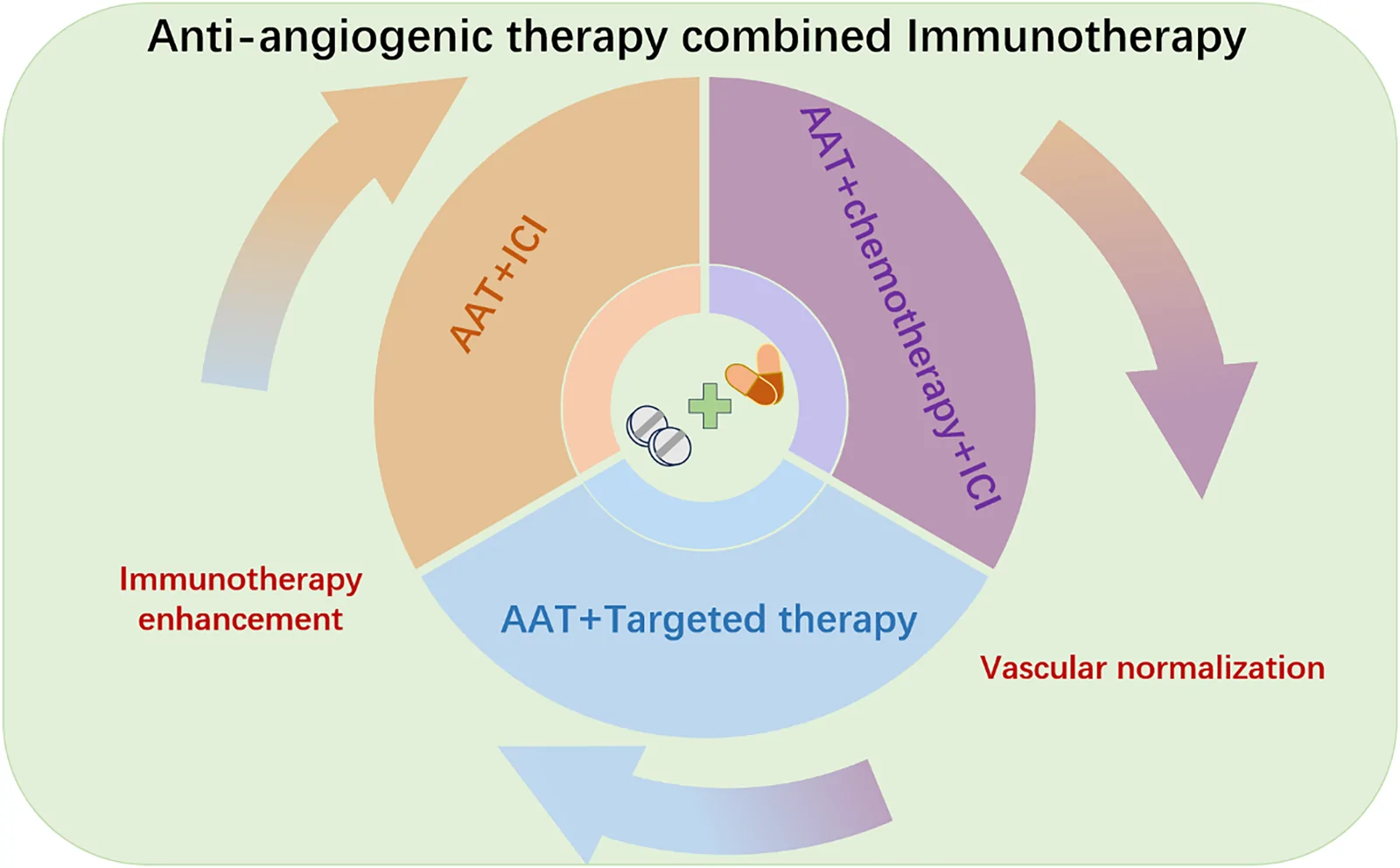
Therapeutic combinations centered on vascular normalization. AAT may be paired with immune checkpoint blockade, chemotherapy plus immune checkpoint blockade, or molecularly targeted therapy. The contribution of normalization depends on agent, dose, sequence, and tumor context and should be demonstrated with pharmacodynamic measurements.

**Table 1:** Representative clinical trials of anti-angiogenic combination strategies in breast cancer.

Combination	Trial	Setting	Regimen	Phase	Primary endpoint and key outcomes	Biomarker signal	Interpretation	Main limitation	Normalization evidence	Ref.
AAT + ICI	SPARK	Locally recurrent or metastaticTNBC 0–3 prior systemic treatment lines; any PD-L1 status Single-arm, multi-cohort study N = 67 enrolled; 61 in the efficacy analysis	Sitravatinib + tislelizumab	II	Primary endpoints: objectiveresponse rate (ORR); also grade ≥3 treatment-related adverse events in Cohort B ORR: 38.1% (70 mg) and 50.0% (100 mg) Median progression-free survival (PFS): 8.2 and 5.4 months, respectively	Expansion of CD8-positive T cells	Preliminary activity; exploratory biomarkers require prospective validation	Single-arm; Chinese cohort; immature OS; biomarker not prospectively validated	Not assessed	(79)
AAT + ICI	LEAP-005	Second- or third-line metastatic or unresectable TNBC Progression after 1–2 prior treatmentlines Single-arm, open-label study N = 31	Lenvatinib + pembrolizumab	II	Primary endpoints: objective response rate (ORR) and safety ORR: 23% by investigators; 32% by blinded independent central review Grade 3 treatment-related adverse events: 52%; one treatment-related death	Exploratory PD-L1 analysis	Supports further randomized testing	Nonrandomized cohort; substantial grade 3 or higher toxicity	Not assessed	(80)
AAT + chemotherapy	E2100	First-line metastatic breast cancer, predominantly HER2-negative No prior chemotherapy for metastatic disease Randomized, open-label study N = 722	Bevacizumab + paclitaxel versus paclitaxel alonel	III	Primary endpoint: progression-free survival (PFS) Median PFS: 11.8 versus 5.9 months No significant overall survival (OS) improvement	Exploratory circulating angiogenic markers	AAT can delay progression without proving durable survival benefit	Vascular normalization was not directly assessed	Not assessed	(81)
AAT + chemotherapy	BEATRICE	Adjuvant treatment after definitive surgery Centrally confirmed operable invasive TNBC Randomized, open-label study N = 2591	Chemotherap y + 1 year of bevacizumab versus chemotherap y alone	III	Primary endpoint: invasive diseasefree survival (IDFS) No significant improvement in IDFS or overall survival (OS)	Exploratory VEGFR2-related analysis	Negative phase 3 evidence limits routine use	Vascular normalization was not directly assessed	Not assessed	(82, 83)
AAT + ICI + chemotherapy	NeoSACT	Neoadjuvant treatment for stage II–III TNBC Single-arm, open-label study N = 29	Anlotinib + sintilimab + chemotherapy	II	Primary endpoint: pathological complete response (pCR; ypT0/is ypN0) pCR: 69.0% 2-year event-free survival (EFS): 92.4%	Lower baseline endothelial-cell abundance and DNA-repair signatures associated with pCR	Strong signal requiring randomized confirmation	Single-arm; small cohort; short follow-up	Not assessed	(4)
AAT + ICI + chemotherapy	FUTURE-C-Plus	First-line advanced immunomodulatory TNBC CD8-positive cells ≥10% by immunohistochemistry Single-arm, open-label study N = 48	Famitinib + camrelizumab + nab-paclitaxel	II	Primary endpoint: investigator- assessed objective response rate (ORR) ORR: 81.3%; median progression-free survival (PFS): 13.6 months	CD8-positive T cells and PD-L1	Supports biomarker-enriched development	Single-arm; selected molecular subtype	Not assessed	(3)
AAT + PARP inhibitor	NCT03075462	Advanced ovarian cancer or TNBC, regardless of BRCA mutation status Prior chemotherapy: 2–4 platinum- based lines for ovarian cancer; ≤2 lines for advanced TNBC Non-randomized, open-label, multicenter study N = 52 (22 with TNBC)	Fuzuloparib + apatinib	I	Primary endpoint: recommended phase 2 dose (RP2D) and tolerability RP2D: fuzuloparib 100 mg twice daily plus apatinib 500 mg once daily TNBC cohort: objective response rate (ORR), 22.7%	Germline BRCA mutation explored	Established feasibility and informed later randomized testing	Small, nonrandomized early-phase cohort	Not assessed	(166)

AAT, anti-angiogenic therapy; EFS, event-free survival; ICI, immune checkpoint inhibitor; OS, overall survival; ORR, objective response rate; pCR, pathological complete response; PFS, progression-free survival; TNBC, triple-negative breast cancer.

### 5.1 Anti-angiogenic therapy plus immune checkpoint blockade

KEYNOTE-355 established the activity of pembrolizumab plus chemotherapy in PD-L1-positive advanced TNBC, but it did not test an anti-angiogenic agent and therefore provides contextual rather than direct evidence for vascular targeting (74). Direct clinical evidence comes from smaller studies. In the single-arm phase 2 ATRACTIB trial, atezolizumab, paclitaxel, and bevacizumab produced a median progression-free survival of 11.0 months and an objective response rate of 63% in first-line advanced TNBC, most of which was PD-L1-negative (75). The signal is encouraging, but the absence of a randomized control means that the specific contribution of bevacizumab—and any claim that it overcomes PD-L1 negativity—remains unproven. Similar caution applies to other phase 2 combinations summarized in **Table 1** (76–80).

The breast cancer experience with bevacizumab illustrates why endpoints and comparators matter. In metastatic HER2-negative disease, adding bevacizumab to chemotherapy improved response rate or progression-free survival in some trials but did not establish a consistent overall-survival advantage. In early TNBC, BEATRICE did not improve invasive disease-free or overall survival. These results do not refute vascular normalization; rather, they show that anti-VEGF activity, chemotherapy delivery, vascular normalization, and durable immune control are not interchangeable outcomes (81–83). Normalization evidence was classified according to the measurements reported in each study: Demonstrated, direct paired or longitudinal evidence of improved vascular function, with supportive structural evidence where available; Inferred, indirect vascular findings compatible with normalization without direct functional confirmation; and Not assessed, normalization was not specifically evaluated or sufficient evidence was not reported. Clinical efficacy outcomes and immune-cell changes alone were not considered proof of vascular normalization. Not assessed should not be interpreted as evidence that normalization did not occur.

For HER2-positive breast cancer, the ECOG-ACRIN E1105 randomized phase III trial showed that adding bevacizumab to chemotherapy combined with trastuzumab did not significantly improve progression-free survival or overall survival, indicating that clinical benefit of anti-VEGF therapy in this subtype has not yet been established (84). Notably, there are ongoing clinical studies evaluating trastuzumab or T-DM1 in combination with immune checkpoint inhibitors (ICI) in HER2-positive disease; however, these trials assess the combination of HER2-targeted therapy with ICI, rather than anti-angiogenic therapy with ICI, and thus cannot serve as direct evidence for the benefit of AAT–ICI (85, 86). In HR-positive/HER2-negative breast cancer, the JCOG1919E/AMBITION randomized phase III study directly compared paclitaxel plus bevacizumab versus a triplet regimen of paclitaxel, bevacizumab, and atezolizumab. The addition of atezolizumab did not significantly improve progression-free survival, and although there was a numerically favorable trend in overall survival, current data are insufficient to draw definitive conclusions (87). Therefore, compared to TNBC, clinical evidence supporting AAT–ICI in both HER2-positive and HR-positive/HER2-negative breast cancers remains limited, and its use should be considered an investigational strategy rather than an established treatment paradigm.

Agent heterogeneity is equally important. Bevacizumab neutralizes VEGF-A, whereas apatinib, anlotinib, lenvatinib, sitravatinib, and famitinib inhibit different combinations of VEGFRs and other kinases. Their effects on pericytes, fibroblasts, myeloid cells, and tumor cells may differ substantially. Describing these regimens collectively as ‘AAT plus ICI’ can obscure the mechanism responsible for benefit or toxicity. A multitarget inhibitor may produce immune effects through TAM or tumor-cell kinases that are independent of vascular normalization. Future reports should link pharmacodynamic findings to the actual target profile and exposure of the tested agent rather than inferring a VEGF-class effect (74–80). Importantly, evidence of target inhibition or reduced angiogenic signaling alone should not be considered equivalent to demonstration of vascular normalization.

### 5.2 Anti-angiogenic therapy, chemotherapy, and immune checkpoint blockade

Chemotherapy can release tumor antigens and induce immunogenic cell death, while vascular remodeling may improve intratumoral drug distribution and alter selected suppressive cell populations (53, 88–90). In the single-arm phase 2 NeoSACT trial, neoadjuvant anlotinib, sintilimab, and chemotherapy achieved a pathological complete response rate of 69.0% and a 2-year event-free survival rate of 92.4% (4). The exploratory associations between baseline endothelial-cell abundance, DNA-repair signatures, and pathological response do not establish treatment-induced vascular normalization. Accordingly, normalization evidence for NeoSACT is classified as Not assessed in **Table 1** because the reported analysis did not establish longitudinal improvements in vascular structure or function. These data support further randomized testing; they do not establish superiority over contemporary chemoimmunotherapy or identify the contribution of each component.

The neoadjuvant setting is attractive because tissue can be sampled before and during therapy and pathological response provides an early efficacy endpoint. Nevertheless, pCR is not a perfect surrogate for long-term outcome in every subtype or regimen. Single-arm pCR rates are particularly difficult to interpret when chemotherapy backbones, stage distribution, PD-L1 prevalence, and assessment methods differ from historical cohorts. Residual cancer burden, event-free survival, and toxicity-related treatment completion should accompany pCR. Mechanistic studies should compare baseline and early on-treatment tissue, rather than relying only on surgical specimens collected after months of therapy, when responding tumors may contain little viable tissue. Randomized window-of-opportunity studies should incorporate paired measurements of perfusion, permeability, vessel maturity, endothelial integrity, and immune-cell localization to distinguish true vascular normalization from nonspecific anti-angiogenic effects (3, 4). Such designs would provide a more direct test of whether an AAT-induced vascular-normalization window contributes to subsequent immunotherapy sensitivity.

### 5.3 Anti-angiogenic therapy plus targeted therapy

PARP inhibition is clinically active in germline BRCA1/2-mutated, HER2-negative metastatic breast cancer. BRCA-associated genomic instability and angiogenic signaling provide a rationale for combining PARP and VEGF-pathway inhibition (91–93). In the phase 3 FABULOUS trial, fuzuloparib plus apatinib improved median progression-free survival compared with chemotherapy and with fuzuloparib alone in patients with germline BRCA1/2 mutations (6). This randomized evidence supports the combination in the studied population, although tolerability, access, and applicability beyond germline BRCA-mutated disease require separate evaluation.

Partner selection should follow breast cancer biology. In germline BRCA1/2-mutated disease, PARP inhibition has a clear synthetic-lethal rationale, and hypoxia-related homologous recombination effects provide an additional but less clinically established reason to combine with Anti-angiogenic therapy. In HER2-positive disease, vascular targeting would need to add value to highly active HER2-directed antibodies, antibody–drug conjugates, and tyrosine kinase inhibitors. In hormone receptor-positive disease, endocrine resistance, PI3K–AKT signaling, and a relatively low-inflammatory microenvironment may be more important than checkpoint blockade alone. These settings should not be combined in one efficacy claim. Trials should define whether AAT is intended to improve drug delivery, intensify DNA-repair stress, or enable immune entry, and select endpoints and biomarkers accordingly (6, 91–93).

Representative trials are summarized in **Table 1**.

### 5.4 Limitations, resistance, and toxicity

Resistance can arise through compensatory FGF, PDGF, Ang-2/Tie2, and hypoxia-related pathways, as well as recruitment of suppressive myeloid cells after prolonged VEGF blockade (25, 94–96). Tumor-intrinsic loss of antigen presentation and induction of alternative checkpoints, including LAG-3 and TIGIT, can further limit ICI activity (97). The existence of these escape routes argues for pharmacodynamic sampling and adaptive treatment schedules rather than uninterrupted maximal VEGF inhibition.

Resistance is likely to be evolutionary rather than static. Early VEGF blockade may improve perfusion, followed by selection of co-opted vessels, activation of FGF or Ang-2 pathways, and recruitment of suppressive myeloid cells. Tumor cells may simultaneously lose antigen presentation or acquire alternative checkpoint programs. A baseline biopsy cannot capture this sequence. Serial analysis should distinguish primary resistance, in which normalization never occurs, from adaptive resistance after a transient biological response. These states may require different interventions: changing dose or schedule for failed normalization, versus targeting a newly dominant angiogenic or immune pathway after initial benefit. Without temporal data, adding more agents empirically risks increasing toxicity without addressing the actual escape mechanism (25, 94–97).

Toxicity constrains combination dosing. Clinically important AAT-related events include hypertension, proteinuria, bleeding, thrombosis, impaired wound healing, and agent-specific hepatic or gastrointestinal effects; severe but uncommon complications require prompt recognition (69). ICIs can cause dermatitis, colitis, hepatitis, pneumonitis, and endocrinopathies (98). Overlapping toxicity, particularly when chemotherapy or a multitarget tyrosine kinase inhibitor is added, should be characterized prospectively and weighed against incremental benefit.

Clinical implementation also requires attention to sequence. Starting Anti-angiogenic therapy before chemotherapy or ICI may create a lead-in period for perfusion improvement, but an excessive lead-in could permit over-pruning. Concurrent initiation is operationally simple yet makes pharmacodynamic attribution difficult. Intermittent dosing could preserve the window and reduce cumulative toxicity, although rebound angiogenic signaling is a concern. Surgical patients require planned interruption because of wound-healing risk, and patients with uncontrolled hypertension, recent thrombosis, or bleeding risk may not be suitable for some regimens. Trials should report dose intensity, interruptions, corticosteroid exposure, and reasons for discontinuation, because these variables affect both vascular and immune outcomes. Patient-reported symptoms and quality of life are particularly important when a modest progression-free-survival gain is achieved at the cost of chronic toxicity (69, 98).

## 6 Biomarkers of the vascular–immune interface

Because vascular normalization is transient and heterogeneous, candidate biomarkers may characterize a baseline state, a treatment-induced biological change, or a spatial vascular–immune feature. These measurements have distinct evidentiary roles. Prognostic biomarkers are associated with disease outcome without necessarily identifying differential benefit from a particular treatment. Predictive biomarkers identify variation in the relative effect of a specified treatment according to biomarker status; evidence should therefore include an appropriate treatment comparison and assessment of the treatment-by-biomarker interaction. An association with response within a single treatment arm, or statistical significance in one subgroup but not another, does not by itself establish predictive value. Pharmacodynamic biomarkers reflect biological responses to treatment but do not necessarily predict clinical benefit or serve as surrogate endpoints. These roles are not mutually exclusive, but each requires supporting evidence in a defined clinical context. VEGF-pathway measures, PD-L1, Ang- 2/Tie2, high endothelial venules (HEVs), TLSs, and circulating cytokines are plausible candidates, but none currently defines a validated vascular-normalization window in breast cancer (75, 99–101). **Table 2** distinguishes biomarker roles, their clinical context, and the evidence supporting treatment-specific prediction.

**Table 2:** Candidate biomarkers of the vascular–immune interface in breast cancer.

Stage	Candidate biomarker	Biological rationale	Evidence in breast cancer	Current interpretation	Biomarker role	Treatment-by-biomarker interaction evidence	Ref.
Baseline	VEGF-A/VEGFR2	Angiogenic drive and VEGF-associated immune suppression	Exploratory AVADO analysis associated high baseline plasma VEGF-A or VEGFR2 with greater PFS benefit	Exploratory interaction signals; predictive utility and clinical cutoffs remain unvalidated	Candidate predictive biomarkers for bevacizumab benefit; exploratory evidence	Exploratory interactions in AVADO were significant at 7.5 mg/kg but not 15 mg/kg; not validated.	(167)
Baseline	VEGF-C, DLL4, NRP1	Alternative or bypass angiogenic signaling	AVF2119g exploratory analysis reported treatment-associated trends	Hypothesis-generating only	Prognostic/mechanistic; candidate predictive	Exploratory AVF2119g findings were not significant after multiplicity correction; predictive value unconfirmed.	(100)
Baseline	PD-L1	Pre-existing adaptive immune resistance	IMpassion130 supported immune-cell PD-L1 as an ICI-associated marker	Validated only within specific ICI assays and settings; not a marker of added AAT benefit	Predictive for ICI	Evidence concerns ICI benefit; interaction for added AAT benefit not established.	(168)
Baseline	Ang-2/Tie2	Vessel stability, permeability, and myeloid–endothelial signaling	Prognostic and response associations in small or exploratory studies	Requires standardized assay and prospective interaction testing	Prognostic/mechanistic; candidate predictive	Prognostic/response associations; interaction for AAT benefit not established.	(104, 106, 107)
On treatment	Circulating VEGF	Target engagement and evolving angiogenic activity	Serial levels have correlated with response or outcome in several cohorts	Pre-analytical effects and lack of validated time points limit use	Treatment-response/pharmacodynamic	Pharmacodynamic associations; predictive interaction not established.	(115, 131–136)
On treatment	Circulating cytokine panel	Immune activation and compensatory myeloid signaling	Neoadjuvant cytokine changes associated with pCR and CD8-positive T-cell infiltration	Promising pharmacodynamic approach; needs a prespecified panel	Treatment-response/pharmacodynamic	Pharmacodynamic/response associations; interaction for added AAT benefit not established	(136)
Spatial	HEVs and perivascular CD8-positive T cells	Lymphocyte entry versus immune exclusion	HEV density and lymphocyte localization associate with immune activity and prognosis	Standardized multiplex imaging and spatial cutoffs are needed	Spatial prognostic/mechanistic; candidate pharmacodynamic	Spatial/prognostic associations; interaction for AAT benefit not established	(140–146, 155–157)
Spatial	TLS presence and maturity	Local antigen presentation and lymphocyte organization	TLSs associate with immune infiltration, prognosis, and ICI response	Promising but assay and maturity definitions remain inconsistent	Prognostic; candidate predictive	Prognostic/ICI-response associations; interaction for added AAT benefit not established	(147–151, 169, 170)

Prognostic, predictive, and pharmacodynamic biomarkers indicate outcome risk, differential treatment benefit, and treatment-induced changes, respectively. Response associations alone do not establish predictive value.

AAT, anti-angiogenic therapy; Ang-2, angiopoietin-2; DLL4, delta-like ligand 4; HEVs, high endothelial venules; ICI, immune checkpoint inhibitor; NRP1, neuropilin-1; pCR, pathological complete response; PD-L1, programed death-ligand 1; PFS, progression-free survival; TLSs, tertiary lymphoid structures; VEGF, vascular endothelial growth factor; VEGFR2, VEGF receptor 2.

### 6.1 Baseline biomarkers

Baseline VEGF-A/VEGFR, PD-L1, and Ang-2/Tie2 measures capture different biological dimensions: angiogenic drive, adaptive immune resistance, and vessel stability (52, 102–107). Their prognostic associations do not automatically imply predictive value. A predictive biomarker must demonstrate a treatment-by-biomarker interaction in an appropriately controlled study.

#### 6.1.1 VEGF-A and VEGFR2

Tumor VEGF-A and endothelial VEGFR2 have been associated with microvessel density, aggressive disease, endocrine response, and breast cancer subtype (102, 108–111). VEGF signaling may also correlate with PD-L1 and other immune features (112, 113). However, tissue heterogeneity, platelet release of VEGF, assay differences, and inconsistent cutoffs limit the use of VEGF-A or VEGFR2 alone for selecting Anti-angiogenic therapy. At present, these markers are best considered biological and prognostic measures with unconfirmed predictive utility.

Analytical validity is a prerequisite for clinical interpretation of VEGF measures. Serum VEGF is strongly influenced by platelet degranulation, while plasma values depend on anticoagulant, processing time, centrifugation, storage, and freeze–thaw cycles. Tissue immunohistochemistry varies with antibody specificity, fixation, scoring compartment, and the distinction between tumor-cell and stromal expression. Soluble VEGFR2 may reflect systemic endothelial effects rather than tumor-specific target dependence. These variables can produce apparently conflicting associations even when the underlying biology is similar. Biomarker studies should use standardized operating procedures, blinded analysis, assay reproducibility metrics, and prespecified cutoffs. A statistically significant correlation within one treatment arm is not enough; prediction requires evidence that the marker modifies the relative benefit of the experimental regimen (99, 102, 108–113).

#### 6.1.2 PD-L1 expression

PD-L1 is the most clinically implemented immune biomarker in TNBC, but its interpretation depends on assay, antibody clone, scoring algorithm, specimen type, and treatment setting (98, 114–118). IMpassion130 linked immune-cell PD-L1 expression to benefit from atezolizumab plus nab-paclitaxel, whereas pembrolizumab programs use combined positive score in relevant settings (115, 116). These are ICI biomarkers; they should not be assumed to predict the added value of Anti-angiogenic therapy. Endothelial or macrophage PD-L1 remains investigational (117, 118).

PD-L1 illustrates the difficulty of transferring a validated biomarker across regimens. Different assays measure tumor cells, immune cells, or a combined score, and thresholds are linked to particular drugs and clinical settings. Archival primary-tumor tissue may not represent a recently treated metastasis, and chemotherapy can alter both PD-L1 and immune-cell composition. In addition, PD-L1 is a dynamic marker of interferon exposure as well as an immune-evasion mechanism. For an AAT-containing regimen, the relevant question is not simply whether PD-L1 predicts ICI activity, but whether vascular targeting adds benefit within PD-L1-defined groups. That question requires an appropriate control arm and a formal interaction test. Endothelial PD-L1 is conceptually distinct from routine tumor or immune-cell scoring and should not be conflated with approved companion diagnostics (114–118).

#### 6.1.3 The angiopoietin–Tie2 axis

Tie2 integrates signals from angiopoietin-1 and angiopoietin-2 and contributes to endothelial survival, maturation, and permeability (119–124). Circulating Ang-2 and Tie2 have shown prognostic or exploratory associations with progression and pathological response in breast cancer (104, 125, 126). Sample sizes are small, cutoffs are not standardized, and no prospective evidence establishes the axis as a treatment-selection biomarker for AAT–ICI combinations.

Ang-2/Tie2 measurements may complement VEGF because they reflect vessel destabilization and endothelial–myeloid interactions. However, the biological meaning of circulating Ang-2 depends on context: it can mark endothelial activation, inflammation, vascular burden, or systemic comorbidity. Tie2 is expressed by endothelial cells and selected monocyte/macrophage populations, so a blood measurement may integrate several compartments. The ratio or joint trajectory of Ang-2, Ang-1, and soluble Tie2 could be more informative than a single baseline value, but such models require assay harmonization and external validation. Importantly, prognostic association with aggressive disease does not establish that Ang-2-high tumors preferentially benefit from a Tie2- or VEGF-directed combination (104, 119–126).

### 6.2 On-treatment biomarkers

On-treatment biomarkers may be more informative than a single baseline measurement because they can capture target engagement, immune remodeling, and the emergence of resistance (115, 127–130). These measures should primarily be regarded as treatment-response or pharmacodynamic biomarkers unless they are prospectively shown to identify differential treatment benefit. For example, a decline in circulating VEGF after AAT may demonstrate target engagement or altered angiogenic signaling, but it does not establish that patients with a greater VEGF decline derive greater benefit from AAT than from an appropriate control. The central challenge is therefore to define sampling times, biologically meaningful thresholds, and prespecified analytical procedures that precede clinical outcome and can be reproduced across regimens.

#### 6.2.1 Dynamic changes in circulating VEGF

Serial circulating VEGF measurements have been associated with disease control, pathological response, and survival in several breast cancer cohorts (131–134). Interpretation is difficult because serum concentrations are influenced by platelet count and activation, specimen handling, and treatment-induced release, and because no standardized change threshold or sampling interval has been validated (135). Plasma- and serum-based assays should not be treated as interchangeable.

#### 6.2.2 Circulating cytokine profiles

In a randomized neoadjuvant study of HER2-negative breast cancer, treatment altered multiple circulating cytokines, and later levels of VEGF-A, IFN-γ, TNF-α, IL-4, and other mediators differed according to pathological response and CD8-positive T-cell infiltration (136). Such panels may capture broader immune–vascular remodeling, but infection, inflammation, batch effects, and multiple-testing risk require prespecified assays and independent validation.

Dynamic biomarker analysis should account for pharmacological perturbation. A rise in circulating VEGF after receptor blockade may reflect feedback or reduced receptor-mediated clearance rather than treatment failure. Similarly, decreases in inflammatory cytokines may indicate reduced tumor burden, immune suppression, or resolution of treatment-related inflammation. Absolute change, percentage change, slope, and time-to-peak answer different questions. Sampling immediately after dosing may capture acute pharmacology, whereas later sampling may reflect tissue remodeling. Studies should therefore anchor time points to dose and biological hypothesis, correct for multiple comparisons, and validate a selected signature in an independent cohort. Exploratory heat maps of dozens of cytokines are useful for discovery but should not be presented as ready-to-use clinical tests (127–136).

### 6.3 Spatial biomarkers

Spatial biomarkers ask whether lymphocytes can cross the vascular interface and reach tumor cells, rather than merely counting cells or measuring bulk expression (137, 138). HEVs and TLSs may mark organized immune recruitment, whereas endothelial inhibitory ligands and perivascular lymphocyte accumulation may indicate exclusion (139–142). These features are biologically attractive but require standardized segmentation, cell definitions, and clinically feasible imaging workflows.

Spatial analysis can separate three clinically different phenotypes: immune desert, vascular or stromal exclusion, and immune-infiltrated tumor. An immune desert lacks substantial lymphocytes and may require antigen-priming strategies; an excluded tumor contains lymphocytes at the margin or around vessels and is the most direct candidate for trafficking-focused intervention; an infiltrated tumor may be limited by checkpoint signaling or other suppressive mechanisms. Bulk TIL or gene-expression scores can blur these distinctions. Quantitative spatial metrics—such as distance from CD8-positive cells to perfused vessels or tumor cells, the fraction of vessels bordered by lymphocytes, and the organization of HEVs and TLSs—could provide a more mechanistically aligned classification. Reproducibility across regions and observers remains a major obstacle (137–142).

#### 6.3.1 High endothelial venules

HEVs are specialized postcapillary venules that support lymphocyte entry. In breast cancer, higher HEV density has been associated with greater lymphocyte infiltration and favorable outcome, and experimental vascular interventions can induce HEV-like vessels (143–146). Their predictive value for AAT-containing therapy is not established, and reproducible staining and scoring criteria are needed.

#### 6.3.2 Tertiary lymphoid structures

TLSs contain organized B-cell, T-cell, and dendritic-cell compartments and can support local antigen presentation and lymphocyte activation (147, 148). Their presence and maturation have been associated with prognosis and response to immunotherapy in breast cancer and other tumors (148–151). Because TLS definitions and maturity scores vary, prospective studies should distinguish prognostic association from treatment-specific prediction.

#### 6.3.3 Endothelial PD-L1

Endothelial cells can express PD-L1, providing a plausible inhibitory signal at the point of lymphocyte extravasation (59, 152–154). Direct breast cancer data remain limited, and endothelial PD-L1 is not captured reliably by routine tumor-cell or immune-cell scoring. Multiplex imaging with validated endothelial markers will be required before this feature can enter clinical stratification.

#### 6.3.4 Perivascular localization of CD8-positive T cells

Perivascular accumulation of CD8-positive T cells without penetration into tumor nests is a spatial phenotype of immune exclusion (77, 155–157). A fall in the perivascular-to-intratumoral gradient during treatment could provide evidence of improved trafficking, but this proposal requires standardized distances, tissue landmarks, and serial sampling.

### 6.4 Integrated multi-omics biomarkers

Integrated genomic, transcriptomic, proteomic, and metabolomic models may better characterize vascular–immune states, but overfitting, batch effects, and small sample sizes limit their translation (158–161). We propose four sequential stages: (1) discovery, identifying candidate features and signatures; (2) analytical validation, establishing assay accuracy and reproducibility using standardized procedures and locked analytical pipelines; (3) clinical validation, confirming performance in independent cohorts and testing treatment-by-biomarker interactions for predictive applications; and (4) clinical utility, demonstrating that biomarker-guided care improves patient outcomes or provides meaningful clinical net benefit. External validation alone does not establish clinical utility. Most current signatures remain exploratory and are not ready for routine clinical use.

Multi-omics models should be developed with the same discipline expected for other clinical prediction tools. Feature selection, missing-data handling, batch correction, and model tuning must be confined to a training set. Performance should then be tested in an external cohort with a clearly defined endpoint. Reporting only discrimination is insufficient; calibration, decision-curve analysis, and comparison with simpler clinical variables are needed. Because treatment selection is the goal, the strongest design includes both experimental and control arms and evaluates whether the model predicts differential treatment benefit. Models derived only from responders within a single-arm study are response classifiers, not validated predictive biomarkers (158–161).

### 6.5 Spatial transcriptomic signatures

Spatial transcriptomics can map endothelial, stromal, and immune programs while preserving tissue architecture (137, 162–165). Reported colocalization of lymphocyte-rich regions with vascular-stability and antigen-presentation programs supports the concept of a spatial vascular–immune niche. However, spatial transcriptomic biomarkers remain largely exploratory in breast cancer. Their clinical implementation is constrained by tissue requirements, sampling heterogeneity, assay cost, analytical complexity, effective spatial resolution, and cross-platform reproducibility. Before routine clinical use, candidate spatial signatures will require standardized tissue acquisition, predefined analytical pipelines, independent validation, and demonstration of clinical utility.

Spatial transcriptomics adds resolution but also introduces sampling and analytical constraints. Most platforms analyze a limited tissue area, and the effective resolution ranges from multicellular spots to near-single-cell measurements. Deconvolution may misassign endothelial, pericyte, fibroblast, and myeloid transcripts when these cells are tightly apposed around vessels. Necrotic and low-RNA regions may also be underrepresented, even though they are biologically relevant to hypoxia and immune exclusion. A clinically transferable workflow should therefore define tissue acquisition and region-of-interest selection in advance, combine spatial transcriptomics with vascular and immune immunofluorescence and histopathological annotation, and use standardized computational pipelines. Where possible, spatial findings should be validated using an orthogonal method or independent cohort rather than relying solely on discovery-platform data. Validation should focus on a small number of biologically interpretable spatial relationships rather than an unfiltered catalog of differentially expressed genes (137, 162–165).

No single candidate currently measures the normalization window with sufficient analytical and clinical validity. The most credible development strategy is a prespecified composite that combines a vascular-state measure, a spatial trafficking measure, and a dynamic pharmacodynamic readout, tested prospectively against a treatment interaction rather than response alone (**Table 2**). Such a composite should ultimately be tested prospectively in randomized treatment settings to determine whether it identifies differential benefit from AAT-containing regimens rather than simply correlating with response or prognosis. Until these steps are completed, integrated vascular–immune signatures should be considered exploratory and hypothesis-generating rather than routine clinical biomarkers.

## 7 Discussion and future directions

The vascular–immune interface helps explain why anatomical vessel density alone is an inadequate therapeutic target. Abnormal perfusion, endothelial anergy, hypoxia, and suppressive myeloid niches jointly restrict immune access and function. Anti-angiogenic therapy can reverse part of this state, but normalization is a dose- and time-dependent biological response rather than an inherent property of every anti-angiogenic regimen.

Clinical evidence in breast cancer is promising but not yet definitive for most AAT–ICI combinations. Randomized phase 3 evidence supports fuzuloparib plus apatinib in germline BRCA1/2-mutated, HER2-negative metastatic disease, whereas several AAT–ICI and triplet studies are single-arm or phase 2. Contextual ICI trials such as KEYNOTE-355 should not be presented as direct evidence for vascular targeting. Likewise, negative bevacizumab trials show that improved progression-free survival or response does not necessarily translate into overall-survival benefit.

A definitive trial architecture would separate mechanism from efficacy. A short Anti-angiogenic therapy lead-in could be randomized against no lead-in, with imaging and paired tissue collected before ICI or chemotherapy begins. The main treatment comparison would then test the full combination against an appropriate standard regimen. Such a design could determine whether early normalization occurs, whether it predicts subsequent immune entry, and whether the addition of AAT improves clinical outcome. Dose-ranging should be biological rather than based solely on maximum tolerated exposure. Biomarker hypotheses, sampling windows, and analysis thresholds should be registered prospectively. Although this design is more demanding than a single-arm combination study, it is far more likely to identify a reproducible therapeutic window and a population that truly benefits.

Progress now depends on trials that measure the proposed mechanism. Lead-in designs should define the onset and duration of normalization using serial imaging, blood, and—where feasible—tissue. Randomization should separate the contribution of AAT from that of chemotherapy and ICI, and biomarker analysis should test treatment interactions using prespecified cutoffs. Safety must be integrated with pharmacodynamic dosing rather than treated as a secondary consideration. Until such evidence is available, vascular–immune biomarkers should be described as exploratory, and claims that Anti-angiogenic therapy converts a ‘cold’ tumor into a ‘hot’ tumor should remain conditional rather than categorical.

Three priorities emerge. First, the field needs harmonized definitions of vascular normalization that combine function, structure, and immune trafficking. Second, subtype- and site-specific studies must replace broad assumptions that all breast tumors share one vascular phenotype. Third, clinical development should favor parsimonious combinations with a measurable mechanism over increasingly complex regimens assembled from preclinical plausibility alone. A successful vascular–immune strategy will not be defined by maximal vessel destruction or by the largest possible biomarker panel. It will be defined by a reproducible biological change, achieved at a tolerable dose, that improves the relative benefit of a partner therapy in a prospectively identified group of patients.

Minimum reporting standards would make studies more comparable. Reports should specify the anti-angiogenic target profile, dose intensity, treatment sequence, chemotherapy backbone, prior ICI exposure, metastatic sites, and the timing of every biospecimen relative to dosing. Vascular endpoints should distinguish perfusion, permeability, density, pericyte coverage, hypoxia, and immune-cell distance rather than combining them into a single qualitative label. Biomarker analysis should report assay platform, compartment, cutoff derivation, missing samples, multiplicity adjustment, and whether the hypothesis was prespecified. For single-arm trials, authors should avoid attributing clinical activity to vascular normalization unless a direct pharmacodynamic change accompanies response. For randomized trials, the key analysis is a treatment-by-biomarker interaction, not a within-arm association. These standards would reduce retrospective overinterpretation and allow negative studies to inform dose and mechanism rather than being dismissed as failures of an entire therapeutic concept.

The likely clinical path differs by subtype. In TNBC, the most immediate opportunity is to determine whether vascular targeting adds benefit to an active chemoimmunotherapy backbone and whether an excluded spatial phenotype identifies that benefit. In HER2-positive disease, the relevant question is whether improved perfusion or immune access can enhance HER2-directed antibodies or antibody–drug conjugates without unacceptable overlapping toxicity. In hormone receptor-positive disease, vascular intervention may need to be paired with endocrine or pathway-directed therapy and evaluated in tumors with demonstrable angiogenic or exclusion features rather than in unselected populations. Brain and liver metastases deserve dedicated cohorts because their native vascular architecture and immune environment differ markedly from those of primary breast tumors. A subtype- and site-specific development program is less convenient than a universal combination strategy, but it is more consistent with the biological heterogeneity that has limited prior anti-angiogenic trials.
